# Determining the footprint of breeding in the seed microbiome of a perennial cereal

**DOI:** 10.1186/s40793-024-00584-3

**Published:** 2024-06-17

**Authors:** Kristina Michl, Christophe David, Benjamin Dumont, Linda-Maria Dimitrova Mårtensson, Frank Rasche, Gabriele Berg, Tomislav Cernava

**Affiliations:** 1 Institute of Environmental Biotechnology, Graz University of Technology, Graz, 8010 Austria; 2 Department of Agroecosystems, Environment and Production, ISARA, 23 rue Jean Baldassini, Lyon Cedex 07, 69364 France; 3 Plant Sciences Axis, Crop Science lab, ULiege - Gembloux Agro-Bio Tech, Gembloux, B- 5030 Belgium; 4 Department of Biosystems and Technology, Swedish University of Agricultural Sciences, P.O. Box 103, Lomma, Alnarp Sweden; 5 Institute of Agricultural Sciences in the Tropics (Hans-Ruthenberg-Institute), University of Hohenheim, 70593 Stuttgart, Germany; 6 International Institute of Tropical Agriculture, P.O. Box 30772-00100, Nairobi, Kenya; 7 Leibnitz-Institute for Agricultural Engineering, 14469 Potsdam, Germany; 8 Institute for Biochemistry and Biology, University of Potsdam, 14476 Potsdam, Germany; 9 School of Biological Sciences, Faculty of Environmental and Life Sciences, University of Southampton, Southampton, SO171BJ UK

**Keywords:** Perennial grain, Seed microbiome, Endophytes, Plant breeding, Amplicon sequencing

## Abstract

**Background:**

Seed endophytes have a significant impact on plant health and fitness. They can be inherited and passed on to the next plant generation. However, the impact of breeding on their composition in seeds is less understood. Here, we studied the indigenous seed microbiome of a recently domesticated perennial grain crop (Intermediate wheatgrass, *Thinopyrum intermedium* L.) that promises great potential for harnessing microorganisms to enhance crop performance by a multiphasic approach, including amplicon and strain libraries, as well as molecular and physiological assays.

**Results:**

Intermediate wheatgrass seeds harvested from four field sites in Europe over three consecutive years were dominated by *Proteobacteria* (88%), followed by *Firmicutes* (10%). *Pantoea* was the most abundant genus and *Pantoea agglomerans* was identified as the only core taxon present in all samples. While bacterial diversity and species richness were similar across all accessions, the relative abundance varied especially in terms of low abundant and rare taxa. Seeds from four different breeding cycles (TLI C3, C5, C704, C801) showed significant differences in bacterial community composition and abundance. We found a decrease in the relative abundance of the functional genes *nirK* and *nifH* as well as a drop in bacterial diversity and richness. This was associated with a loss of amplicon sequence variants (ASVs) in *Actinobacteria*, *Alphaproteobacteria*, and *Bacilli*, which could be partially compensated in offspring seeds, which have been cultivated at a new site. Interestingly, only a subset assigned to potentially beneficial bacteria, e.g. *Pantoea, Kosakonia*, and *Pseudomonas*, was transmitted to the next plant generation or shared with offspring seeds.

**Conclusion:**

Overall, this study advances our understanding of the assembly and transmission of endophytic seed microorganisms in perennial intermediate wheatgrass and highlights the importance of considering the plant microbiome in future breeding programs.

**Supplementary Information:**

The online version contains supplementary material available at 10.1186/s40793-024-00584-3.

## Background

Plant seeds harbor diverse endophytes whose composition is determined by plant genotype, environment, and management practices [1]. Plant breeding has been recognized as an important driver of plant-associated microbial diversity [2], but to what extent and how domestication affects seed microbiomes is less understood. Despite the increasing knowledge about the beneficial role of the plant microbiome, it has been neglected in most breeding programs. This prompted researchers to propose that breeding programs should consider the manifold interactions of plants and soil microorganisms [3, 4]. Accordingly, the microbiomes of plants that are bred for specific agronomic traits, like seed size and shattering, may undergo substantial changes [5]. In addition, seeds in particular should be considered in future breeding efforts due to their role as transgenerational vehicles of beneficial and facultative pathogenic endophytes. Seeds serve, together with soil, as an initial reservoir of microbes for emerging seedlings and are important for plant development and health [6]. It was previously shown that both soil and seed bacterial communities influence the assemblage of wheat seedling microbiomes and that specific bacteria are vertically transmitted across plant generations in tomato plants [7–9]. Various studies focused on entangling factors influencing the seed microbiome assemblage, and it was shown that plant genotype as well as environmental conditions are involved in this process [10]. However, research focusing on the seed microbiome of cereal plants is still underrepresented compared to studies targeting below-ground compartments [11].

Perennial grain cropping systems provide a complement to annual cereals for improved environmental sustainability in grain production. The continuous cover and large root systems of perennial plants provide a solution for various environmental problems associated with intensive annual cultivation, including nutrient leakage and soil erosion. The recently domesticated perennial intermediate wheatgrass [*Thinopyrum intermedium* (Host) Barkworth & D.R. Dewey; trademarked Kernza®] has gained international attention for grain production [12]. The grain yield of intermediate wheatgrass is well below that of annual wheat, and breeding programs were implemented to increase the harvest [13]. While initial breeding efforts focused on intermediate wheatgrass as a forage crop, the Land Institute (TLI) established a promising domestication program in the early 2000s that prioritized grain production. In the initial six cycles of this breeding program, selection was based on phenotypic traits, while starting from the seventh cycle TLI shifted to genotypic selection [13].

Information on the positive ecosystem services of intermediate wheatgrass is increasingly available, however, knowledge of the microbiome of this crop is scarce. A limited number of studies addressed the microbiome of intermediate wheatgrass, targeting only below-ground compartments [14, 15]. Perenniality and management influence the communities of soil nematodes, earthworms and bacteria associated with intermediate wheatgrass and the plants generally sustain more complex food webs compared to annual wheat [14, 16]. Furthermore, the deep roots of wheatgrass were shown to enrich potential plant-beneficial bacteria [15]. To date, no study has been published on endophytic microbial communities in intermediate wheatgrass. Acquiring a more profound understanding of the indigenous seed microbiota in plants untouched by intensive breeding can contribute to the characterization of a robust and healthy microbiome [17].

In this study, we analyzed 16S rRNA gene amplicons of wheatgrass seed endophytes, harvested from four different fields in three European countries (Sweden, Belgium, and France) over three consecutive harvest years to answer the following research questions: (i) which bacterial endophytes are present in intermediate wheatgrass seeds?; and (ii) does a core microbiome exist in the seeds, and do successive seed generations exhibit similarity over consecutive years? If not so, (iii) are there specific effects related to harvest years and fields?; and (iv) does breeding have an effect on the seed microbiome? If so, (v) does this effect persist into the next generation? The intriguing characteristics, such as its perennial nature and short domestication period, make it an especially interesting candidate to address the aforementioned research questions.

## Materials and methods

### Intermediate wheatgrass seed collection

Intermediate wheatgrass [*Thinopyrum intermedium* (Host) Barkworth & D.R. Dewey; trademarked Kernza®] seeds from breeding cycle TLI C3 were collected from three countries in Europe: Sweden (55°40′0″N, 13°5′0″E), Belgium (50°34′0″N, 4°41′0″E) and France (45°34′5″N, 5°15′58″E for ST. Marcel site and 45°63′29″N, 5°25′72″E for Maubec site), in either two or three harvest years (2020, 2021, 2022). Furthermore, seeds from different breeding cycles (TLI C3, C5, C704, C801) were obtained from the Land Institute (TLI) in Kansas, USA (38.7684° N, 97.5664° W; parental/seeding material) and from three breeding cycles (TLI C5, C704, C801) in 2022 from Belgium (50°34′0″N, 4°41′0″E; harvested/offspring material). The seeds from cycle C3 were not sown on the new field in Belgium, therefore, no offspring material for C3 was available. The intermediate wheatgrass breeding program was established by TLI in the early 2000s. While C3 and C5 were produced by breeding with phenotypic selection, the cycles C704 and C801 were genotypically selected [13]. Furthermore, the number of parents used to generate the different cycles was drastically reduced between the phenotypic (80 and 70 parents for C3 and C5, respectively) and genotypic (5 and 14 parents for C704 and C801, respectively) selection. The whole field was harvested as one batch and a subset of the seeds was obtained for the subsequent analysis. The samples were stored dry and dark until further use. Further information on the utilized seeds can be found in the Supplementary Material (Table S1).

### Surface sterilization and seed germination

Wheatgrass seeds were surface sterilized with 4% sodium hypochlorite for 5 min and rinsed with sterile distilled water 5 times. Finally, 100 µl of the final rinse was spread on Nutrient-Agar plates and incubated at room temperature for one week to confirm successful surface sterilization. The surface-sterilized seeds were germinated at room temperature in the dark for 24 h on sterile, water-soaked cotton pads. Five germinated seeds per sample were combined and in total ten replicates for each sample type were processed. The surface sterilized seeds were stored at -20 °C until further use.

### DNA extraction and 16S rRNA gene fragment sequencing

Seeds were disrupted with mortar and pestle and total genomic DNA was extracted using the DNeasy PowerSoil Kit (Qiagen, Valencia, CA, USA) following the manufacturer’s instructions and stored at -20 °C until further use. The V3-V4 region of the 16S rRNA gene was amplified using the universal barcoded primers 515f (5′-GTGYCAGCMGCCGCGGTAA‐3′) and 806r (5′‐GGACTACNVGGGTWTCTAAT‐3′) [18]. Peptide nucleic acid clamps (PNA) were added to the PCR mix to inhibit the amplification of host plastid and mitochondrial 16S rRNA genes [19]. PCR amplifications were performed in a total volume of 25 µl and in two technical replicates using the 2x KAPA Taq Ready Mix (Kapa Biosystems, USA), 1.5 µM PNA mix, 0.2 mM of each primer, PCR-grade water, and 0.5 µl template DNA. The following cycling conditions were applied: 96 °C for 3 min, 30 cycles of 95 °C for 30 s, 78 °C for 5 s, 54 °C for 30 s, 72 °C for 20 s, and a final extension at 72 °C for 30 s. Technical replicates were pooled and samples from the sampling years 2020 and 2021 were combined in equimolar concentrations while samples from the sampling year 2022 were included in a separate library. After purification (Wizard SV Gel and PCR Clean-Up System, Promega, Madison, WI, USA), the amplicon libraries were sent to Novogene (Cambridge, UK) for library preparation and sequencing on the Illumina NovaSeq platform (2 × 250 bp paired-end reads).

### Bacterial quantification

For quantification of 16S rRNA gene copy numbers, a quantitative real-time PCR (qPCR) was performed using the universal primers 515f-806r. The reaction mix contained 5 µl KAPA SYBR Green (Kapa Biosystems, USA), 0.5 µl of 10 µM primer, 0.15 µl of each pPNA (50 pmol/µl) and mPNA (50 pmol/µl), 2.7 µl PCR-grade water, and 1 µl template DNA (1:50 diluted in PCR-grade water). Fluorescence intensities were quantified with the Rotor-Gene 6000 real-time rotary analyzer (Corbett Research, Sydney, Australia) as described previously [20]. For the determination of *nirK* and *nifH* gene abundance, the reaction mix was adjusted: 5 µl KAPA SYBR Green, 0.5 µl of each primer (10 µM) (*nirK*: 5’-ATYGGCGGVCAYGGCGA-3’ and 5’-GCCTCGATCAGRTTRTGGTT-3’; *nifH*: 5’AAAGGYGGWATCGGYAARTCCACCAC-3’ and 5’-TTGTTSGCSGCRTACATSGCCATCAT-3’ [21]), 3 µl PCR-grade water, and 1 µl template DNA (1:50 diluted in PCR-grade water). Temperatures were set as follows for *nirK*: 95 °C for 3 min, 40 cycles of 95 °C for 5 s, 58 °C for 30 s, 72 °C for 5 s and a final melt curve of 72–96 °C. The same temperature setup was used for assessing *nifH* gene copies with the temperature for primer annealing changed to 55 °C. All samples were subjected to two individual qPCR runs with each two technical replicates. The gene copy numbers found in the negative controls were subtracted from the samples [22].

### Bioinformatics and statistical analysis

Cutadapt was used to demultiplex the data and eliminate primer sequences and low-quality reads [23]. Using the DADA2 algorithm [24] in QIIME2 [25] the data was quality filtered, denoised, and chimeric sequences were removed. The obtained representative sequences (amplicon sequence variants (ASVs)) were classified using the SILVA v132 database with the vsearch algorithm [26, 27].

Bacterial community analysis was performed using the package Phyloseq [28] and statistical analysis was performed with R (version 4.3.1) [29] in R studio (version 2023.06.1) [30]. To assess bacterial alpha diversity, the dataset was normalized by random subsampling to 175 reads per sample using the function *rarefy_even_depth* and seed set to 5163. This value was chosen as a trade-off between sequencing depth and retaining a high number of biological replicates. A total of nine samples had to be removed with these settings due to low read numbers. To determine evenness, the Shannon H’ index was divided by the natural logarithm of species richness [31]. Small variations in species detection and sample coverage due to limited sequencing depth can impact alpha diversity metrics, in particular species richness [32]. The restricted sequencing depth of the subsampled dataset can result in an inflated estimation of evenness. The Kruskal-Wallis test was carried out to determine significant differences in bacterial abundance and microbial alpha diversity, based on Shannon H’ index, species richness, and evenness. Groups were compared with pairwise Wilcoxon test and p-values were adjusted using false discovery rate as a correction. For beta diversity analysis, the dataset was subjected to cumulative sum scaling and Bray-Curtis dissimilarity matrices were calculated. Significant differences were assessed using the function *adonis2* (PERMANOVA, 999 permutations) from the package VEGAN [33]. Linear discriminant analysis effect size (LEfSe) implemented in MicrobiomeAnalyst was used to assess differentially abundant bacterial genera or phyla between different breeding cycles [34, 35]. The cut-off for differentially abundant bacterial taxa was set at a LDA score > 2 and *P* > 0.05. The core microbiome members were assessed using the function *core_members* from the package MICROBIOME [36].

For the phylogenetic tree, the subsampled dataset was used and the sequences were aligned using MUSCLE [37]. Subsequently, distance matrices were calculated using the maximum-likelihood algorithm with the MEGA11 software [38] and the phylogenetic tree was visualized using iTOL [39].

## Results

### Community composition, but not diversity, of the seed endophytes is influenced by field site and harvest year

A total of 4,594,964 reads remained after quality filtering and removal of plant-originated sequences (18,924,517). The median number of reads per sample was 5,179 (min = 22, max = 435,665). The reads were assembled to 1,959 ASVs and assigned to 29 bacterial phyla and 407 genera.

**Fig. 1:**
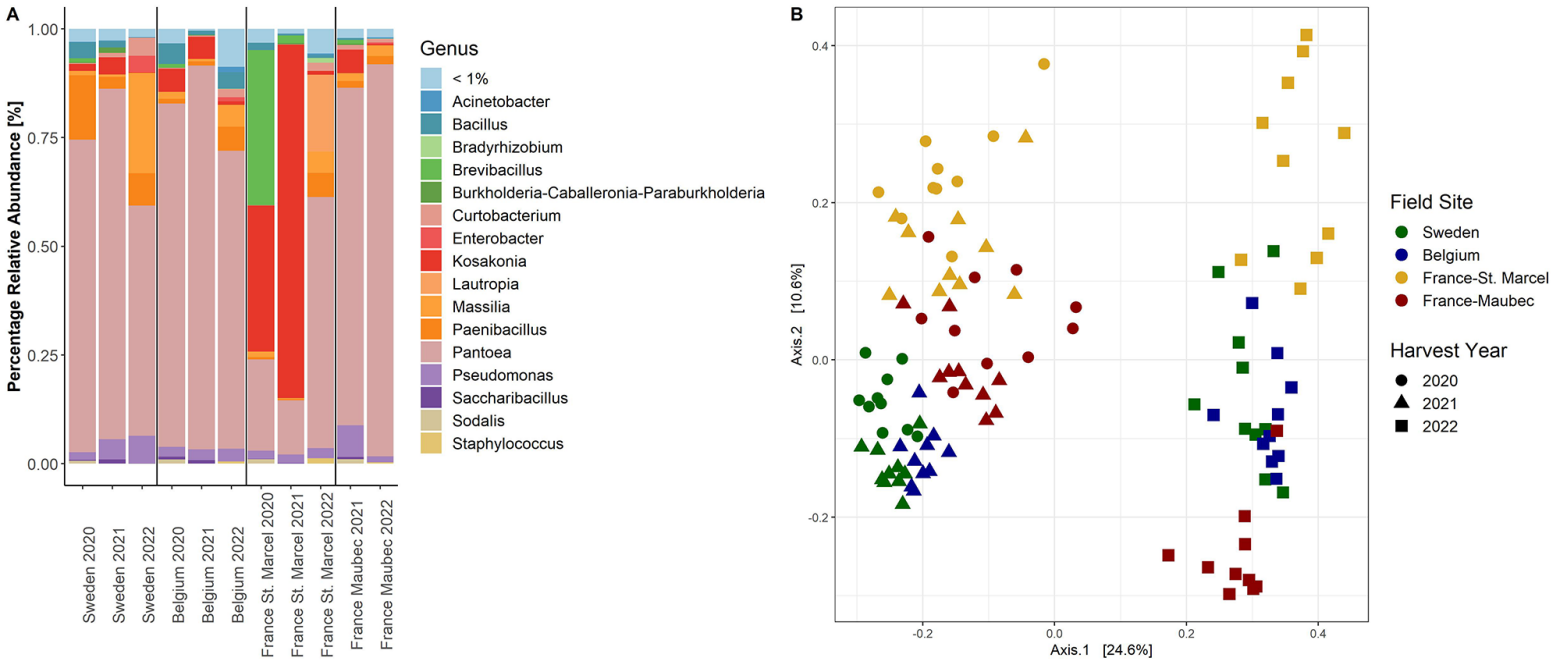
(**A**) Bacterial taxonomic composition of intermediate wheatgrass seed endophytes at genus level. Samples were collected from four different field sites and three harvest years and ten replicates were averaged. The group “<1%” was created from ASVs with a relative abundance lower than 1%. (**B**) Bacterial community composition visualized with a PCoA plot based on Bray-Curtis dissimilarity matrix. The percentage of variation explained by each axis is shown in square brackets

For the general assessment of the intermediate wheatgrass seed microbiome, seeds from four fields with different climatic and soil conditions for three consecutive years were collected. The wheatgrass seed microbiome was dominated by *Proteobacteria* (61.1–96.8%), followed by *Firmicutes* (2.8–38.1%) (Fig. S1). On the genus level, *Pantoea* was the most abundant genus with an average of 63.6%, followed by *Kosakonia* (12.6%) (Fig. 1A). All samples had a similar abundance pattern, except for the samples originating from the field site St. Marcel in France harvested in 2020 and 2021. Compared to the rest of the samples, they were dominated by *Kosakonia* and *Brevibacillus* and to a lesser extent by *Pantoea*. The relative abundance of *Pantoea* was lower in the harvest year 2022 at the sites in Belgium and Sweden compared to the other years, however, at both sites in France the relative abundance was higher.

Alpha diversity analysis revealed that neither harvest year nor field site showed a significant effect on Shannon diversity (mean *H*’: 1.2) or species richness (mean: 12.3) (Fig. S2A-D). However, the combined factor “harvest year and field site” resulted in a significant difference in species richness (*P* = 0.009), but not Shannon diversity, and subsequent pairwise comparisons revealed no further significant results (Fig. S3B).

Contrary to the abundance and diversity, all factors tested significantly influenced the bacterial community composition. Since the samples were sequenced in two batches, this factor was included in the analysis. The factor “batch” overlaps with the factor “harvest year”, since all samples collected in 2022 were in a separate batch, but all field sites were represented in both sequencing libraries. Based on Bray-Curtis dissimilarity, PERMANOVA (adonis2(ps_CSS ~ batch*field_site*harvest_year, permutations = 999)) was performed and “batch” explained 22.6% (*P* = 0.001) of the variation in the bacterial composition, followed by “field site” (12.1%, *P* = 0.001) (Fig. 1B). The factor “harvest year” explained 1.6% (*P* = 0.004) of the variation. To eliminate the potential impact of the sequencing batch, PERMANOVA (adonis2(ps_CSS_20_21 ~ field_site*harvest_year, permutations = 999)) was conducted exclusively with samples harvested in 2020 and 2021. The factor “field site” explained 38.7% (*P* = 0.001) of the variation, followed by “harvest year” (7.1%, *P* = 0.001) (Fig. S4).

### The effect of breeding on the intermediate wheatgrass seed microbiome

To investigate potential implications of breeding on the seed microbiome, seeds from four different breeding cycles (C3, C5, C704, C801) were analyzed. Seeds originating from TLI in Kansas from breeding cycles C5, C704, and C801 were sown in Belgium in 2021 and harvested one year later (2022). Both the parental material and the offspring seeds were subjected to 16S rRNA gene fragment sequencing.

**Fig. 2:**
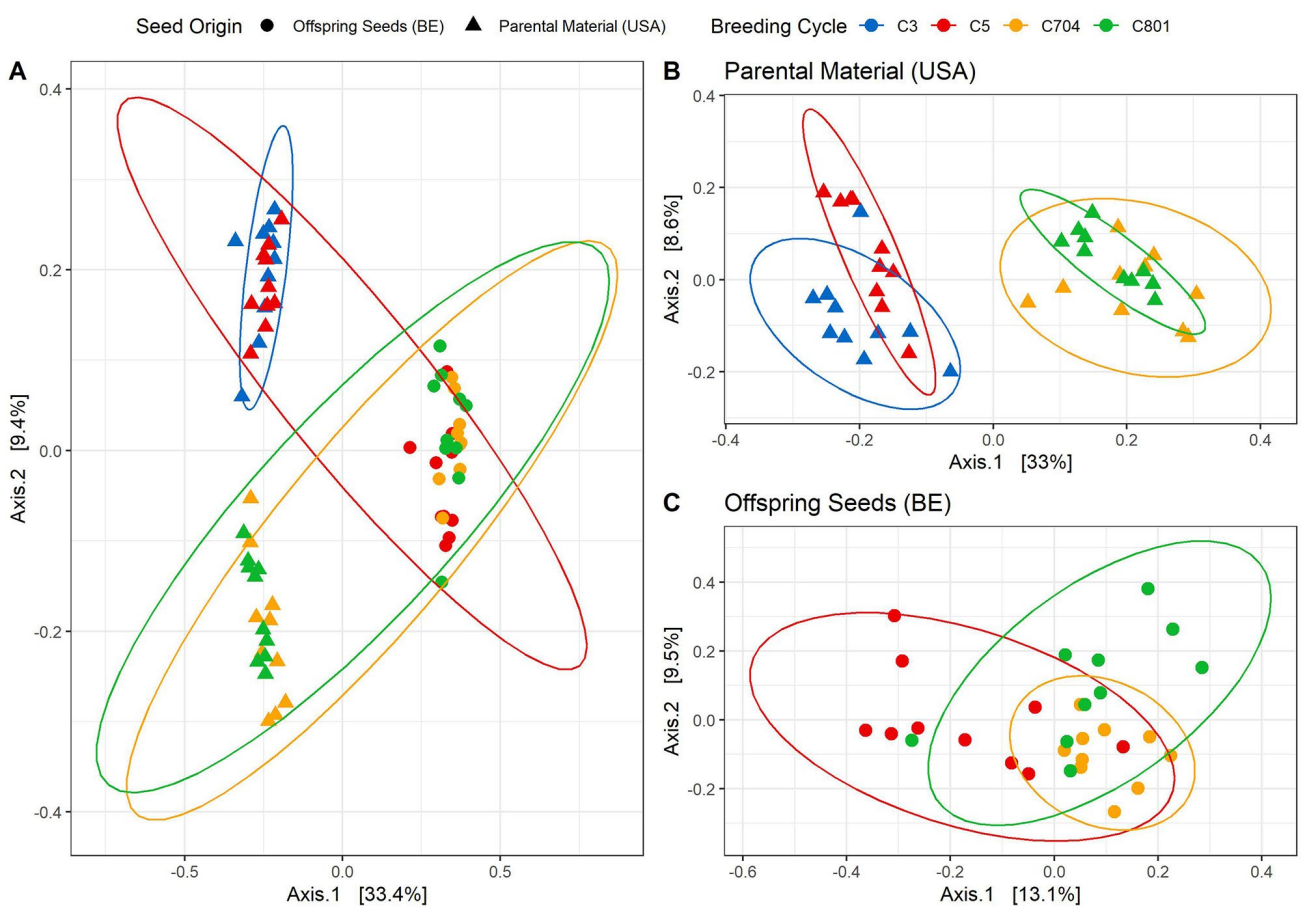
PCoA plots based on Bray-Curtis dissimilarity matrix displaying bacterial community composition of four different breeding cycles (C3, C5, C704, and C801) of intermediate wheatgrass at two different sampling sites with all samples (**A**), and separated in the parental material (**B**) and the offspring seeds (**C**). The percentage of variation explained by each axis is shown in square brackets and significant differences in the bacterial community composition were assessed using PERMANOVA.

A high influence of the factor “field site, harvest year, and/or sequencing batch” on the bacterial community composition was confirmed by PERMANOVA (32.9%, *P* = 0.001) (adonis2(ps_breeding_CSS ~ batch*breeding, permutations = 999)). In addition, a significant influence of the breeding cycle (9.5%, *P* = 0.001) was found (Fig. 2A). Upon separation into the two field sites, a noticeable clustering emerged, particularly for the parental material, delineating a distinct clustering within the earlier (C3 and C5) and later (C704 and C801) breeding cycles (Fig. 2B). For the offspring, a distinct grouping between C5 and C801 was observed, whereas C704 revealed an intermediate clustering (Fig. 2C). The breeding cycle accounted for 38% (*P* = 0.001) of the variation in the community composition for the parental seed material, whereas it only explained 14.7% (*P* = 0.001) in the seeding material.

**Fig. 3:**
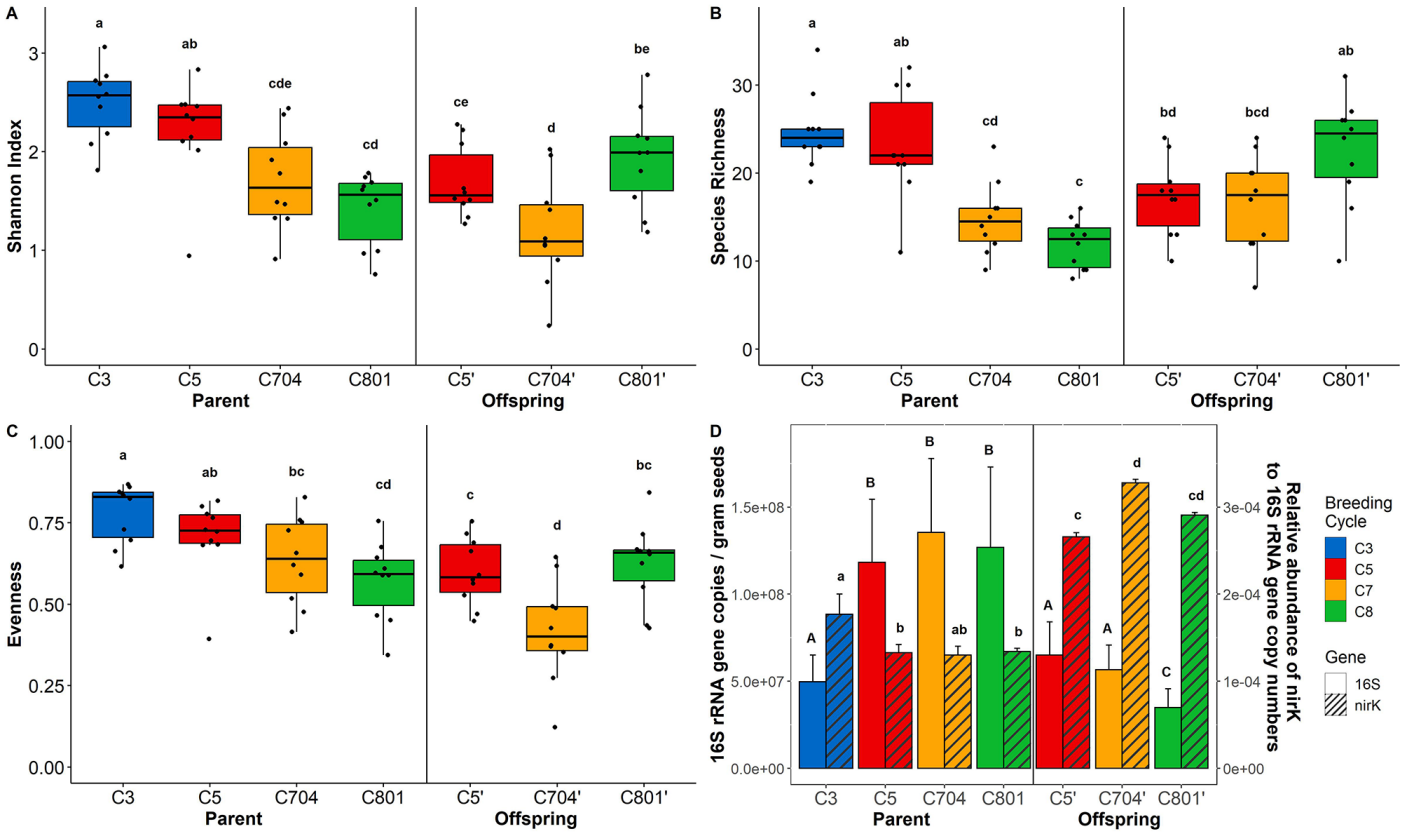
Alpha diversity of the seed microbiome displayed as Shannon H’ (**A**), species richness (**B**), and evenness (**C**). Bacterial abundance per gram seed and relative abundance of *nirK* were assessed with qPCR (**D**). Significant differences (*P* < 0.05) were determined by Kruskal-Wallis pairwise test and are indicated by letters. Uppercase and lowercase letters indicate significant differences between 16S rRNA genes and *nirK* genes, respectively

Calculation of Shannon H’, species richness, and evenness indicated a significant decrease in bacterial diversity with ongoing breeding in the parental material (Fig. 3A-C). For the offspring seeds, no significant difference was found in terms of diversity and evenness between the four breeding cycles. An exception was cycle C704, which was significantly lower compared to cycles C5 and C801 (Fig. 3A, C). Furthermore, bacterial richness and diversity were found to be higher with the number of parents used to generate the different breeding cycles (Fig. S5). A similar trend as for alpha diversity was observed for bacterial abundance, which was estimated by qPCR (Fig. 3D). Due to the high number of reads assigned to plant host DNA, we additionally normalised the 16S rRNA gene copy numbers to the percentage of host DNA inferred from amplicon sequencing (Fig. S10). Although the overall result was similar, the offspring seeds had significantly lower copy numbers than the parental material. The copy numbers of *nirK* and *nifH* genes were highly correlated (*r* = 0.89). For the parental material, 16S gene copy numbers were lower in breeding cycle C3 than in cycles C5/C704/C801, while the relative abundance of *nirK* (Fig. 3D) and *nifH* (Fig. S6) was higher. However, for the offspring seeds, the bacterial abundance was significantly lower in C801 than in C5/C704, while the relative abundance of *nirK* and *nifH* was highest in C704.

**Fig. 4:**
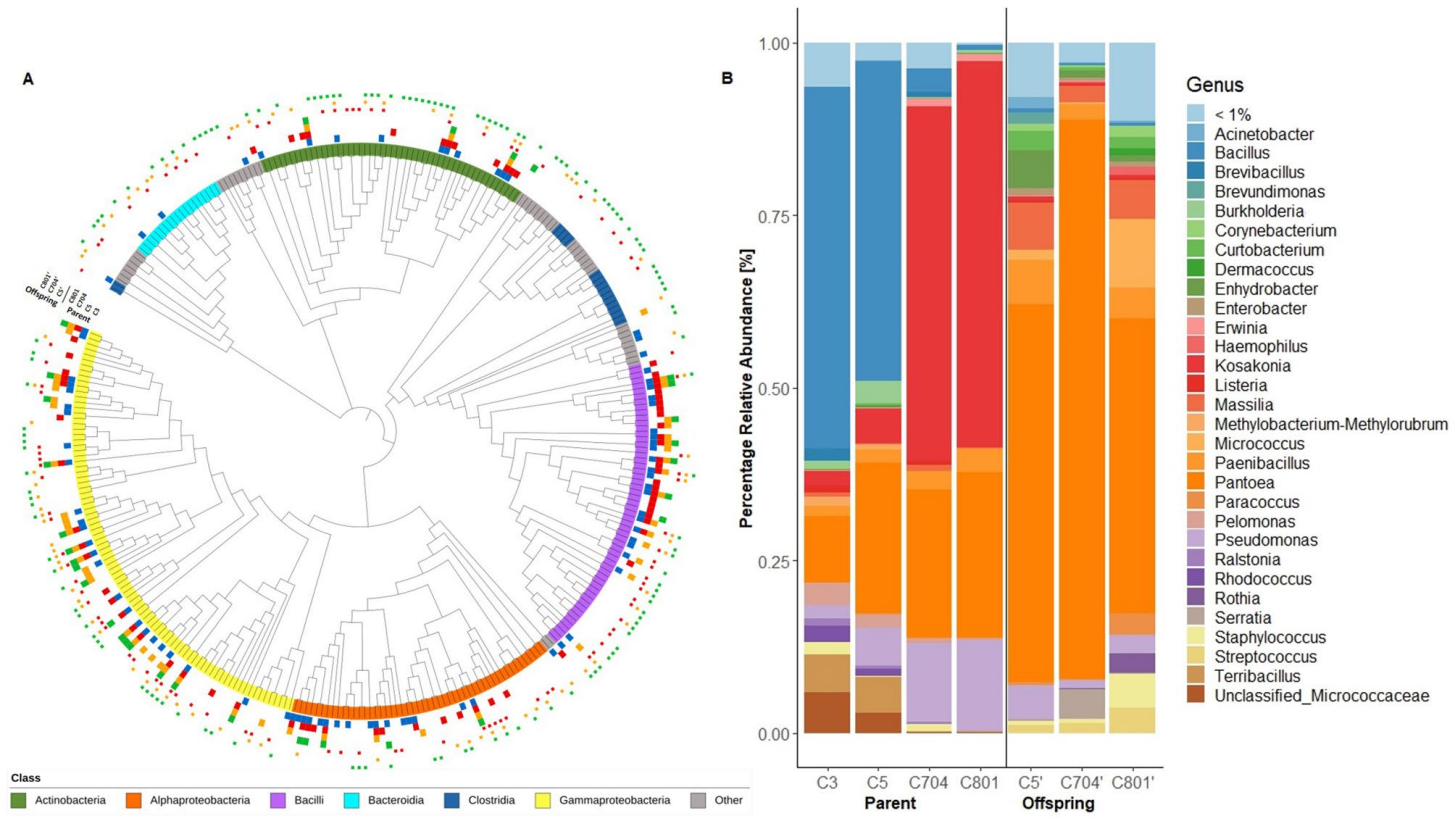
Phylogenetic tree based on ASVs constructed from 16S rRNA gene fragments (**A**). The taxonomy on class level is shown in the inner ring. Ring 2–8 (Parent: C3, C5, C704, C801 and Offspring: C5’, C704’, C801’) indicate the presence of ASVs after random subsampling. Bacterial taxonomic composition of intermediate wheatgrass seed endophytes at genus level (**B**). Samples were obtained for four different breeding cycles (C3, C5, C704, C801) and two field sites. Ten replicates were averaged and the group “<1%” was created from ASVs with a relative abundance lower than 1%

A phylogenetic tree based on the presence and absence of ASVs in the subsampled dataset (*n* = 323) was constructed to assess which bacterial classes were mainly affected by breeding. Thereby, a lower number of ASVs belonging to *Actinobacteria* (*n* = 10, 11, 3, 4; Kruskal-Wallis chi-squared: *P* = 0.000003), *Alphaproteobacteria* (*n* = 16, 13, 5, 5; Kruskal-Wallis chi-squared: *P* = 0.00003), and *Bacilli* (*n* = 23, 25, 16, 7; Kruskal-Wallis chi-squared: *P* = 0.00001) was revealed in the parental material in the later breeding cycles C704 and C801, compared to C3/C5, while ASVs belonging to *Gammaproteobacteria* (*n* = 25, 20, 26, 16; Kruskal-Wallis chi-squared: *P* = 0.2) were less affected (Fig. 4A). Together with a change of ASVs we observed a shift in relative abundances of the affected bacterial classes: the first two breeding cycles (C3, C5) of the parental material were dominated by *Bacilli* (65.7% and 54.2%, respectively), followed by *Gammaproteobacteria* (21.6% and 39%, respectively), while in the later breeding cycles (C704, C801) *Gammaproteobacteria* (mean: 92.6%) was the most abundant class (Fig. S7). These observations were confirmed by LEfSe analysis indicating that four bacterial classes (*Gammaproteobacteria*, *Alphaproteobacteria*, *Bacilli*, and *Actinobacteria*) and 12 bacterial genera were significantly different abundant (Fig. S9, Table S7). On genus level, breeding cycles C3 and C5 of the parental material were dominated by *Bacillus* (52.3% and 46.2%), while *Kosakonia* (51.2% and 55.9%) was detected as the most abundant genus in cycles C704 and C801 (Fig. 4B). *Pantoea* was the second most abundant genus in all four breeding cycles (mean: 19.3%). A higher relative abundance of *Pseudomonas* (1.8–13.2%), *Pantoea* (9.6–24%), and *Paenibacillus* (1.5–3.3%) was detected in the later breeding cycles. On the other hand, the relative abundance of *Terribacillus* (5.4% to < 1%) and rare microbiome members (6.4% to < 1%) was lower.

The offspring seeds were dominated by *Pantoea* (mean: 59.5%) and a similar microbial composition compared to the samples collected in Belgium in 2022 was detected, which, however, differed from the composition of the parental material (Figs. 1C and 4B). *Pantoea* was the predominant genus, followed by *Massilia* (mean offspring: 4.9%, Belgium 2022: 5.1%) and *Paenibacillus* (mean offspring: 4.4%, Belgium 2022: 5.5%). The predominant genera from the parental material, *Bacillus* and *Kosakonia*, were among the rare taxa in the offspring seeds.

### The intermediate wheatgrass core microbiome and its transmission

Core members of the intermediate wheatgrass seed microbiome were defined based on the presence of the ASV in at least 9/10 replicates with a detection threshold of 1/1000. The core microbiome consisted of only one shared ASV, which was assigned to *Pantoea agglomerans*. This ASV was the only core bacterium in the overall dataset and represented 57.1% of the total reads. Furthermore, this ASV showed 100% sequence similarity with a culturable isolate (Table S3). Interestingly, this *P. agglomerans* isolate was highly abundant in all breeding cycles in offspring seeds, but not in the parent generation. This suggests that this strain might play a vital role for the host plants in the new environment in which the seeds have been sown. Different in-vitro bioassays were used to assess lytic enzyme activity and secondary metabolite production. The isolate was able to solubilize phosphate, and produce rhamnolipid, siderophores, and IAA, but did not show proteolytic activity. In total, 38 AVSs were part of the core of individual sample types. While the replicates from Belgium collected in 2022 only shared one core ASV, seeds collected in France (2021) had in total 16 core ASVs. In general, only a small subset of the taxa, yet compromised of abundant ones, was shared between the replicates, while the rest of the taxa varied. Only ten of these ASVs were shared over at least two consecutive years in the same field site or between parent and offspring and assigned to: *P. agglomerans*, *Kosakonia* sp., *Pantoea* sp., *Pseudomonas* sp., *Massilia* sp., *Paenibacillus* sp., *Brevibacillus* sp., *Enhydrobacter* sp., and *Curtobacterium* sp. The other 29 core ASVs were only detected once per field site and were not transmitted to the next generation or shared between the offspring (Table S2).

## Discussion

The seed microbiome in cereals is still sparsely understood and remains understudied in comparison to other plant compartments [11]. The present study is the first to analyze the seed microbiome of Kernza®, a perennial grain crop mainly bred for grain production, while maintaining other relevant ecosystem services [16, 40–42]. It indicates a general prevalence of *Proteobacteria* and *Firmicutes*, and a potential key role of the genus *Pantoea* as a core and inherited component. Given the plant’s recent and well-known breeding history, the specific impact of the breeding progress could be explored in detail.

It was previously discussed by Gutierrez and Grillo [43] that both domestication and breeding influence the plant microbiota, causing changes in the abundance and structure of microbial communities, including microorganisms with potential functional significance. However, there is little evidence that domestication influences microbial diversity [43]. For example, a higher microbial diversity was observed in the seeds of cultivated plant species compared to wild relatives in both wheat [44] and rice [45]. On the other hand, Özkurt et al. [46] described a decrease in diversity in domesticated wheat compared to wild progenitors. However, these studies compared plants that were domesticated thousands of years ago, while the presented study was based on plants that were bred for three to eight cycles, corresponding to a maximum of 30 years of targeted breeding. In the present study, a strong decrease in bacterial diversity and richness, associated with a loss of ASVs in the bacterial classes *Actinobacteria*, *Alphaproteobacteria*, and *Bacilli*, was observed in the course of breeding. Additionally, a shift in the community composition between the earlier two breeding cycles (C3 and C5) and the later ones (C704 and C801) was observed. Interestingly, cycles C3 and C5 were generated via phenotypic selection, while cycles C704 and C801 were bred via genotypic selection [13]. We propose that the phenotype-based selection considers the whole holobiont, which includes the plant and its associated microorganisms [47], as they may influence the phenotype of the plant. Earlier findings indicated that the seed-endophytic *Sphingomonas melonis* has the potential to induce a phenotypic shift in rice plants under pathogen pressure. This shift manifests a transition from a disease-susceptible to a disease-resistant phenotype [48]. However, since it is difficult to differentiate microbiome effects from environmental influences, there is only limited information on the impact of microorganisms on plant phenotypes [49, 50]. In contrast, genotype-based selection disregards the concept of a holobiont, and only considers plant genetic traits. It was further suggested that the focus in previous breeding efforts on purely agronomic traits, such as seed shattering, may have resulted in a loss of unique microorganisms specific to e.g. the hull tissue [51]. Furthermore, cycles C3 and C5 were established from 80 and 70 parents, respectively, while C704 and C801 were derived from only 5 and 14 parents, respectively. This reduction in plant genetic diversity may have subsequently resulted in a decrease in the associated microbiome. Interestingly, when plants from different breeding cycles were grown at a new field site, the offspring had a similar microbiome composition, diversity, and richness. In terms of the decrease in bacterial diversity with ongoing breeding, we propose that plants select only a subset of bacteria with essential functions required in a certain environment. Microbiome (*M*) genes are host genes that control the assembly and structure of microbial communities [52]. A recent study investigating maize plants from over 129 accessions and diverse environments not only showed that the host genetically controls its root microbiome, but also that local adaptions occur [53]. In addition, seeds are a specific habitat for microbes with strong selective pressure. Small changes in the seed environment, like differences in nutrient composition, but also seed size, may affect seed endophytes [43, 50, 54]. Furthermore, it was shown for soybean seeds that storage periods of up to 14 months led to a decrease in diversity and had an influence on the bacterial composition, but without changing relative abundances. The seeds were, independent of storage time and temperature, dominated by *Gammaproteobacteria* [55]. In the seeding material, we observed a higher relative abundance of *Firmicutes* in the earlier breeding cycles, while the later cycles exhibited an increase in *Proteobacteria*, a switch that was previously associated with breeding practices [56]. However, the seeds from the earlier breeding cycles C3 and C5 were stored for 11 and 5 years, respectively, while the seeds from C704 and C801 were only stored for two years. It is possible that the extended storage period may have impacted the bacterial abundance and composition, favouring spore-forming bacteria commonly observed within the phylum *Firmicutes*.

It was previously proposed that the seed microbiome can serve as a functional toolbox, especially in nutrient poor environments, emphasizing the importance of the seed microbiome in plant breeding efforts [57, 58]. Bacteria play a crucial role in the nitrogen (N) cycle by altering the availability of (soil) N for plants [59]. Hence, *nirK* and *nifH* genes were chosen as representatives for this functional group to examine the effects of breeding on the functional microbiome. Interestingly, the 16S rRNA gene copy numbers in the offspring seeds decreased from cycle C5 to C801 and were significantly lower compared to the parental material, though the seeds were substantially younger. Furthermore, the age of the seeds could also explain the increase in copy numbers in the parents. Compared to C704/C801 the seeds from C3 and C5 were 9 and 3 years older, respectively. On the other hand, the relative abundance of *nirK* and *nifH* genes decreased in the parent seeds within the course of breeding but increased in the progeny. This pattern resembles alpha diversity and reinforces the idea of translocating plants after breeding at one field site to another environment to preserve not only taxonomic but also functional diversity. The environmental microbiome is an important source and influences the endophytic microbiome and should therefore be considered in breeding programs. Comparable to the current study, a previous investigation observed similar relative abundance patterns of *nirK* gene copy numbers in wheat seedlings [60]. In adult intermediate wheatgrass roots, however, they were almost 100-fold higher [15]. Though, the relative abundance of *nirK* and *nifH* gene copy numbers decreased over time in wheat and *Brassica* seedlings respectively, suggesting that the sampling timepoint may have a substantial influence on their occurrence [57, 60]. In addition, bacteria can have varying numbers of 16S rRNA gene copies and the use of peptide nucleic acid (PNA) may result in differences in the 16S rRNA gene copy numbers [61, 62]. It should be noted that the high number of reads attributed to host DNA, particularly in the offspring material, could have led to an overestimation of both the 16S rRNA gene copy numbers and the relative abundance of functional genes. It is also important to note that *nirK* and *nifH* are only two of numerous functional genes, making it challenging to discuss the potential for a loss of function through breeding.

The bacterial community composition of the intermediate wheatgrass seeds was dominated by *Proteobacteria*, and especially *P. agglomerans* in most field sites. The latter was further identified as the only core taxon present in all samples. Less abundant constituents of the seed microbiome differed between the samples. It was previously reported, that seed-associated microbial communities often include a low number of highly abundant species, but also a high number of rare taxa [63], which, according to Rezki et al. (2018), may be vulnerable to local extinction. Simonin et al. [6] showed in a comprehensive meta-analysis on the seed microbiome that the majority of seeds was dominated by *Proteobacteria* and that one ASV, assigned to *P. agglomerans*, was conserved in 68.5% of the samples collected from 27 different plant species from all over the world. The *P. agglomerans* isolate obtained in the present study showed plant growth promoting characteristics and rhamnolipid production in vitro, which was previously described for other strains [64, 65]. While diversity and species richness were stable over the years and field sites, both of these factors had a significant impact on the bacterial community composition. It has been previously reported that environmental factors, such as geographical location and harvest year, influence seed microbiome assemblage [10]. In accordance with our results, soil was shown to influence the seed microbiome serving as an important reservoir for microbes that are internalized [8].

Concerning the transmission of bacteria from parental plants to the offspring seeds over one or two generations, we observed that only a limited number of core taxa was shared between the offspring from different years. In addition to the core bacterium *P. agglomerans*, only core taxa assigned to the genera *Paenibacillus*, *Kosakonia*, *Pantoea*, *Pseudomonas*, *Bacillus*, *Massilia*, *Curtobacterium*, and *Brevibacillus* were shared over at least two consecutive years. Therefore, these were identified as potentially vertically transmitted bacteria. However, the other 29 constituents of the various core microbiomes were not inherited by the offspring. It was previously shown for *Brassica* [66] and *Setaria viridis* [8] that most of the seed constituents are acquired by horizontal transfer and that the inheritance rate was low. Furthermore, Morales Moreira et al. [10] discussed that the observed differences between parent and offspring plants reflect environmental conditions. Interestingly, based on the perennial nature of the host plants, we propose that if certain environmental factors, like surrounding soil, remain consistent, this should be reflected in a more stable seed microbiome among the offspring over the years. On the other hand, Mitter et al. [67] showed in wheat that the bacterial strain *Paraburkholderia phytofirmans* PsJN introduced via the flower was internalized into the seed, with plant growth promoting effects. Although only a few taxa were transmitted in the intermediate wheatgrass seeds, they may play an important role in the host plant, especially during early plant development. Building on the hypothesis of Taffner et al. [68] that archaea as late plant colonizers are randomly transmitted or only present in the seed as bystander microorganisms, it is plausible that bacteria, deemed non-essential in early development stages, are transmitted without targeted selection. Notably, a significant alteration in the microbiome was observed in the parent and offspring seeds harvested from different field sites. Despite the diverse microbiome of the parent seeds from different breeding cycles, the offspring exhibited a comparatively consistent composition. A transition from a prevalence of *Bacillus* and *Kosakonia* to *Pantoea* was observed, however, other components appeared to be specific to sample types, as indicated by the limited number of core ASVs. Moreover, the microbiome composition mostly reflected the environmental conditions, as previously found [10]. It was demonstrated that parent and offspring seeds can reveal different bacterial communities. Consequently, a proposition was made that, akin to mammals, which can pass on beneficial microorganisms to their progeny, plants also transfer selected beneficial bacteria to the next generation [7, 69]. The transmission of microorganisms from the mother plant to the offspring and further on to the seedling are critical and complex processes. Hence, a more comprehensive understanding of these processes could open avenues for future advancements in breeding practices [70]. To move beyond taxonomic diversity and address the functional microbiome, additional research combining metagenomics and cultivation-based approaches will be required. Furthermore, it will be important to extend research to other members of the microbiome, such as archaea and fungi, given their potential to establish symbiotic (or pathogenic) associations with their host plants [54].

## Conclusion

The present study provides key insights into the impact of breeding on endophytic bacterial communities in intermediate wheatgrass seeds. The data indicates that breeding substantially affected the diversity, composition, and abundance of the seed microbiome. However, a transfer to a new cultivation site reversed some of the effects in the offspring seeds. This underlines the importance of future breeding programs considering the plant as a holobiont that is closely related to its microbiome. In addition, the environmental microbiome should be considered as an important source for the acquisition of seed endophytes.

### Electronic supplementary material

Below is the link to the electronic supplementary material.

Supplementary Material 1

Supplementary Material 2

## Data Availability

Raw sequencing data for each sample used in this study was deposited at the European Nucleotide Archive (ENA) in the FASTQ format and is available under the Bioproject accession number PRJEB73380.
