# Supplementary material for: Determining the footprint of breeding in the seed microbiome of a perennial cereal
Source: Environ Microbiome. 2024 Jun 17;19:40. doi: 10.1186/s40793-024-00584-3 (PMC11184768; doi:10.1186/s40793-024-00584-3)
Supplement: Supplementary file 2 — Supplementary Material 2 [file 40793_2024_584_MOESM2_ESM.docx]

**Supplementary Information for**:

Determining the footprint of breeding in the seed microbiome of a perennial cereal

Kristina Michl, Christophe David, Benjamin Dumont, Linda-Maria Dimitrova Mårtensson, Frank Rasche, Gabriele Berg, and Tomislav Cernava

**Supplementary Material and Methods**

## Isolation and identification of intermediate wheatgrass seed endophytes

Intermediate wheatgrass seeds originating from Sweden, Belgium, and France from the harvesting year 2021 were subjected to surface sterilization and germination as described above. Approximately 15 germinated seeds were ground in 2 ml 0.9% NaCl and a dilution series up to 10 ^-5^ was plated on LB and R2A and the plates were incubated at room temperature for four and seven days, respectively. Based on their morphological differences, 108 bacterial isolates were further subcultured on LB.

The bacterial isolates were identified based on their 16S rRNA gene sequence. Bacterial genomic DNA was extracted following the Triton method [1]. PCRs, using the universal primer set 27F (5’-AGAGTTTGATCCTGGCTCAG-3’) and 1492R (5’-GGACTACHVGGGTWTCTAAT-3’), were performed as described previously [2] and the PCR amplicons were sequenced (LGC Genomics, Berlin, Germany). The sequences were manually quality filtered using BioEdit [3] and compared against standard databases using the Basic Local Alignment Search Tool [4].

Screening for plant beneficial traits

The production of siderophores and the ability to solubilize phosphate was determined according to Flemer et al. [5]. Furthermore, isolates were tested for rhamnolipid [6], indole-3-acetic acid (IAA) production [7], and proteolytic activity [8].


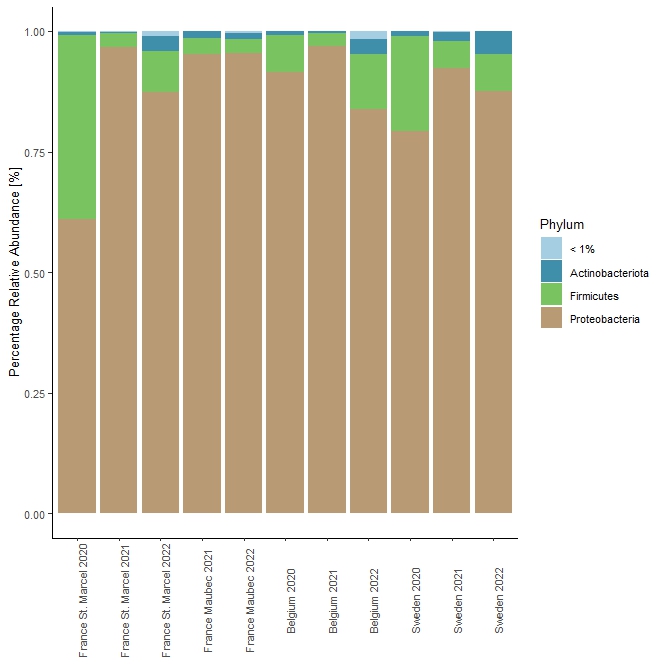


Figure S1: Bacterial taxonomic composition of intermediate wheatrgass seed endophytes at phylum level. Samples were collected from four different field sites and three harvest years and ten replicates were averaged. The group “<1%” was created from ASVs with a relative abundance lower than 1%.


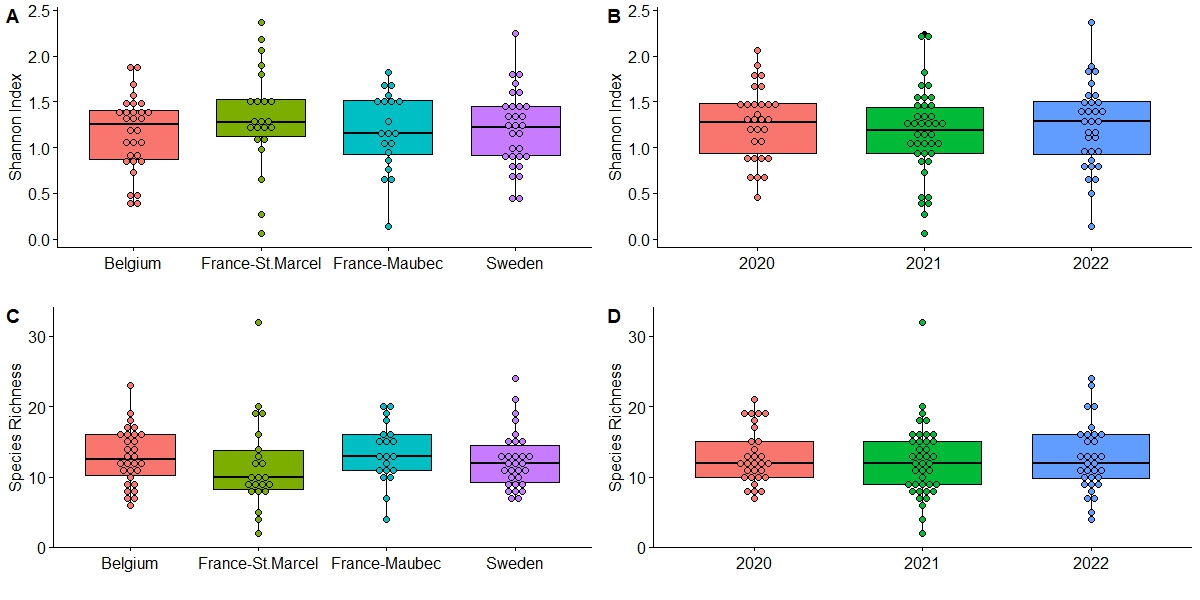


Figure S2: Alpha diversity of seed microbiome displayed as Shannon H' (A, B) and species richness (C, D). Samples were merged according to field-site and harvest year and no significant difference was observable.


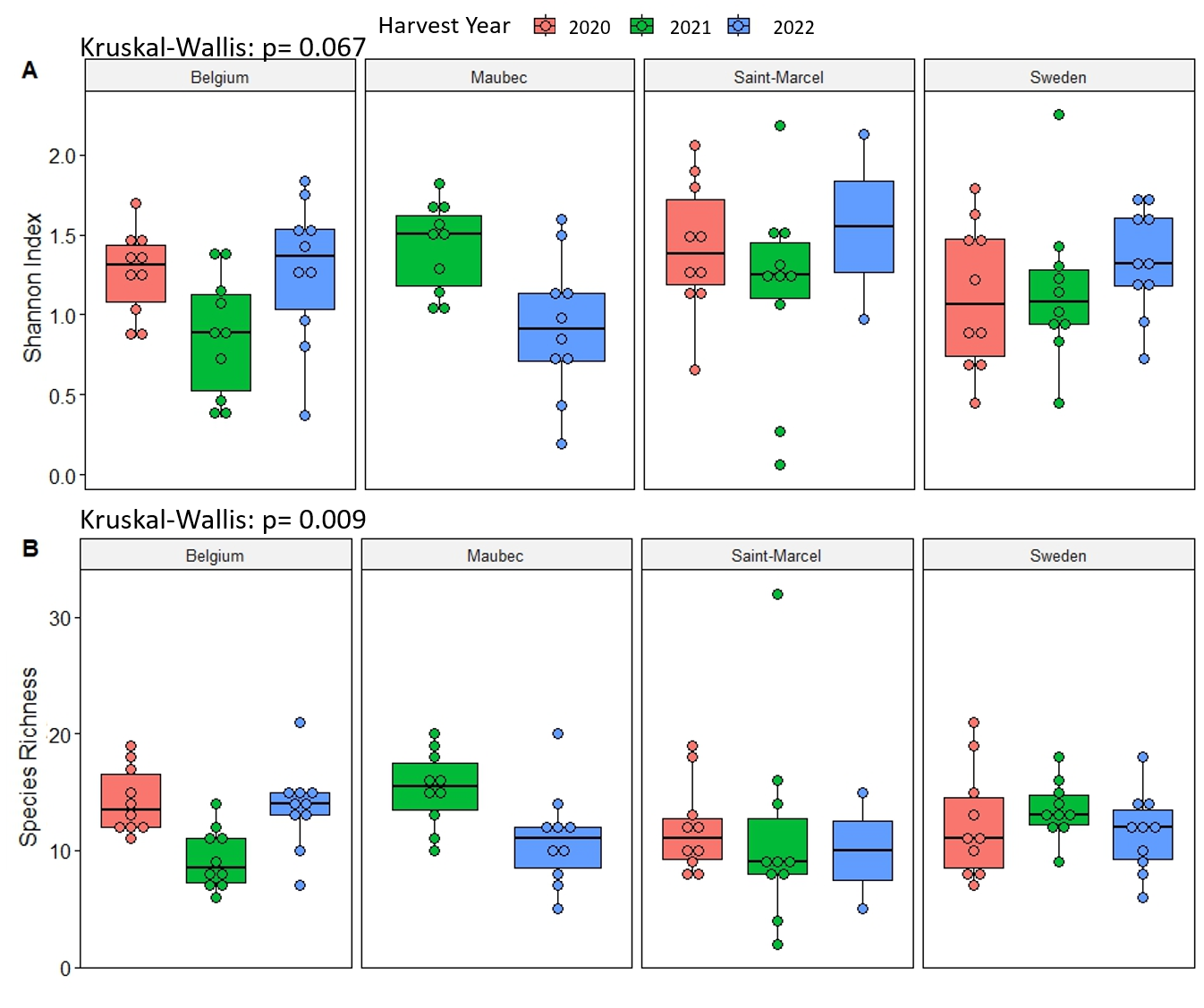


Figure S3: Alpha diversity of intermediate wheatgrass seed microbiome seperated per harvest year and field site and displayed as Shannon H' (A) and species richness (B).


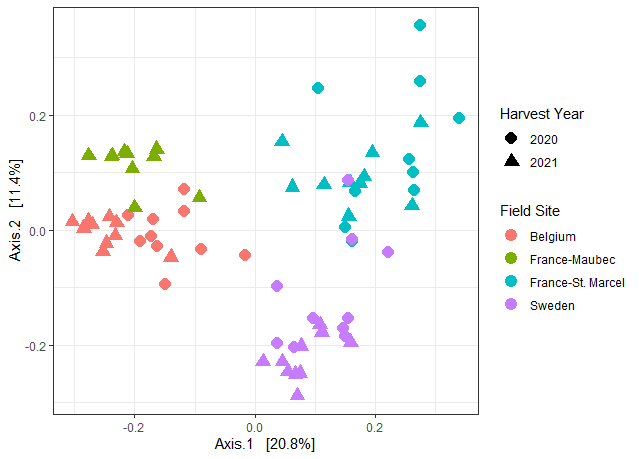


Figure S4: (B) Bacterial community composition from samples harvested in 2020 and 2021 visualized with a PCoA plot based on Bray-Curtis dissimilarity matrix.


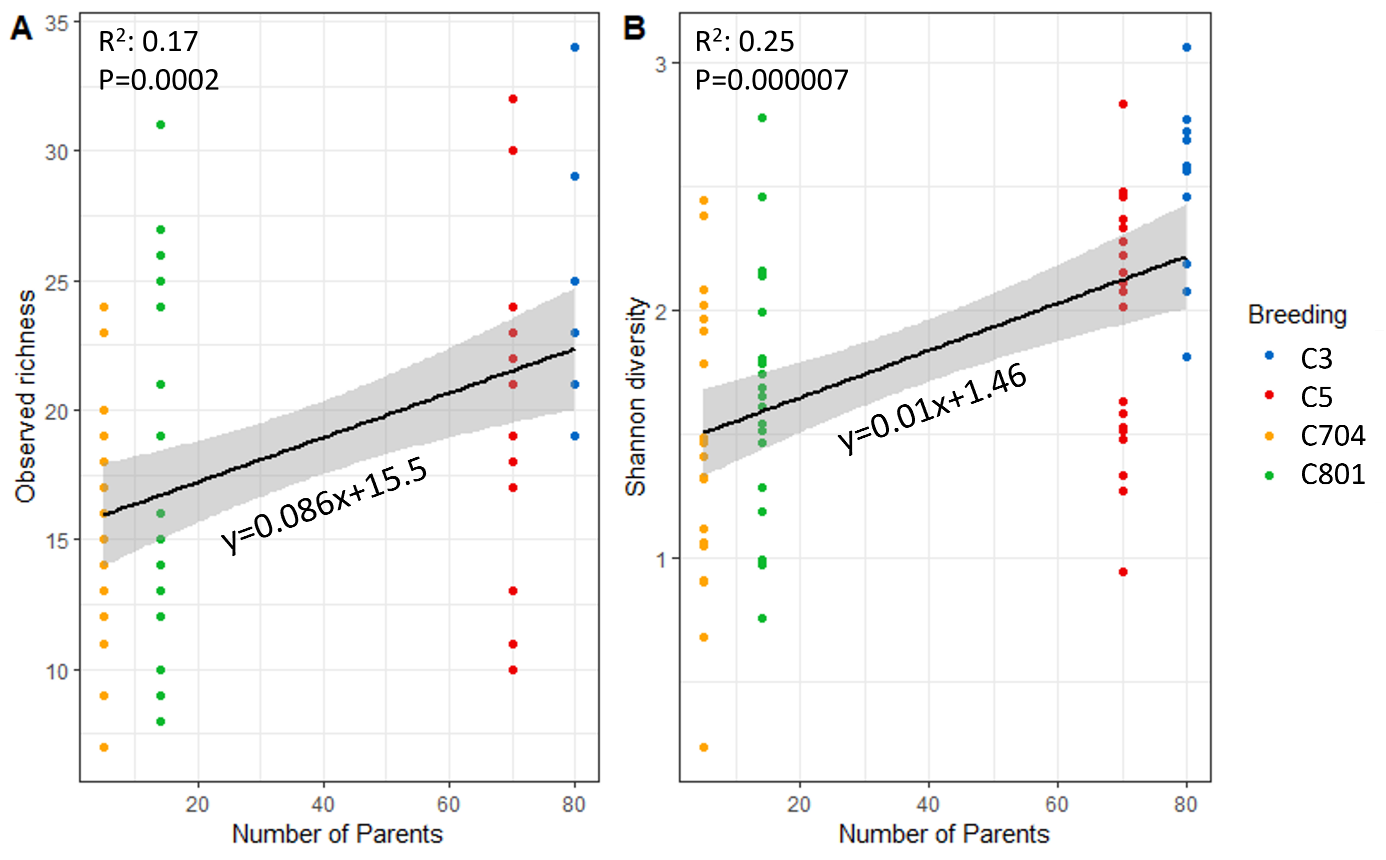


Figure S5: Effect of the number of the parents used to generate the breeding cycle on (A) Observed richness and (B) Shannon diversity, explained by linear models. Two field sites and different harvest years were merged.


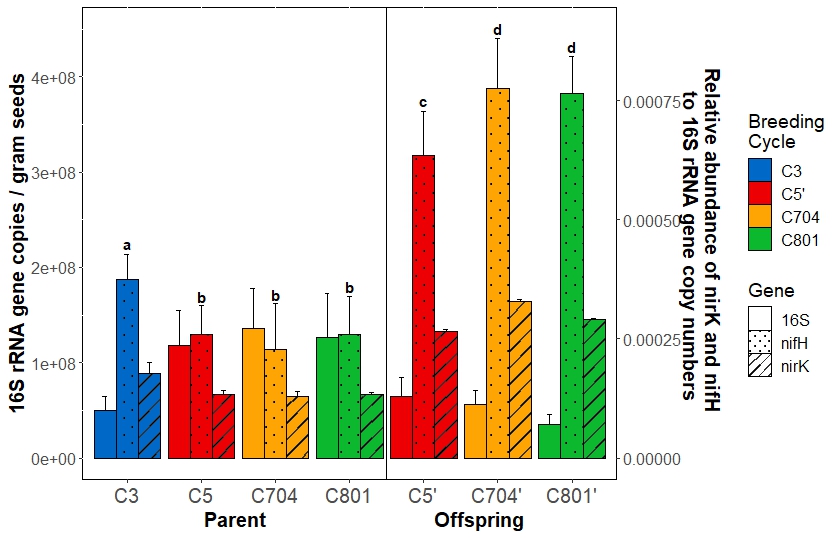


Figure S6: Bacterial abundance per gram seed and relative abundance of nirK and nifH were assessed with qPCR. Significant differences (P< 0.05) were determined by Kruskal-Wallis pairwise test and are indicated by letters.


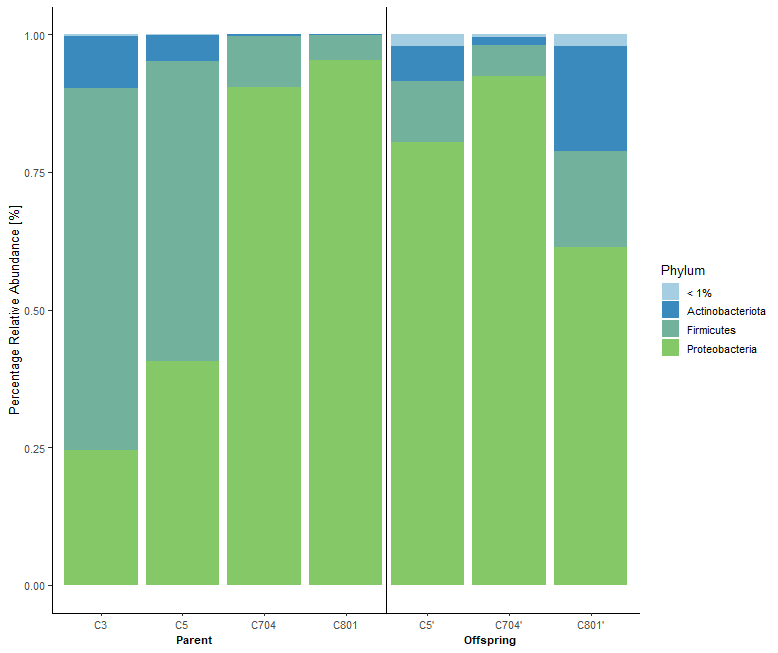


Figure S7: Bacterial taxonomic composition of intermediate wheatgrass seed endophytes at class level. Samples were collected from four different breeding cycles (C3, C5, C704, C801) and two field sites. Ten replicates were averaged and the group “<1%” was created from ASVs with a relative abundance lower than 1%.


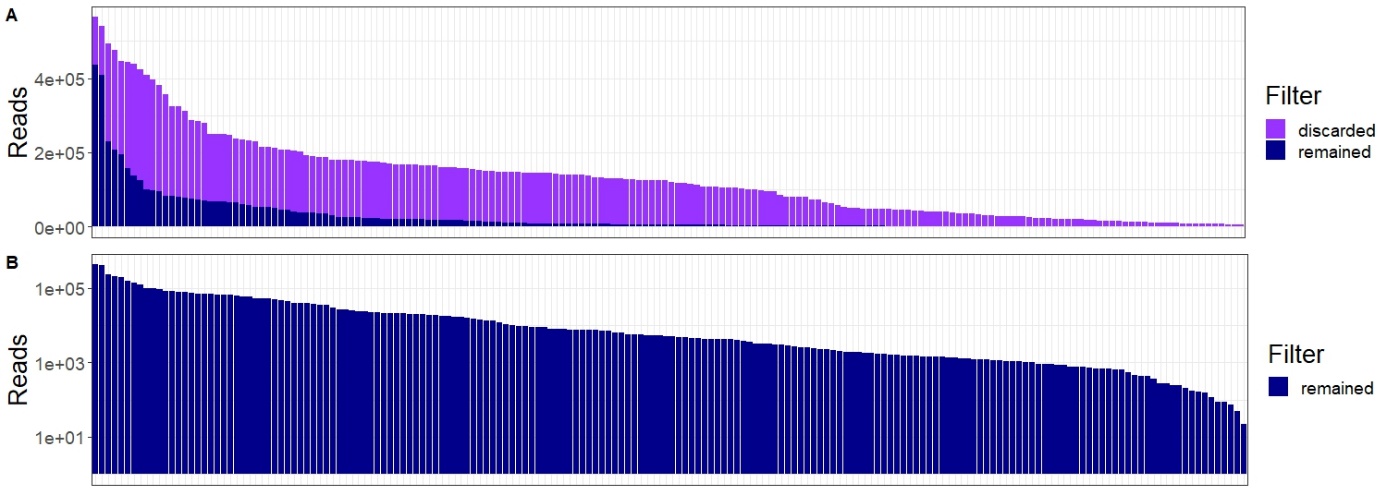


Figure S8: Barplots with the number of reads per sample. (A) Reads which were discarded are shown in purple and reads that are retained for subsequent analysis are depicted in blue. (B) Close-up on the number of reads retained for subsequent analysis on a log-scale.


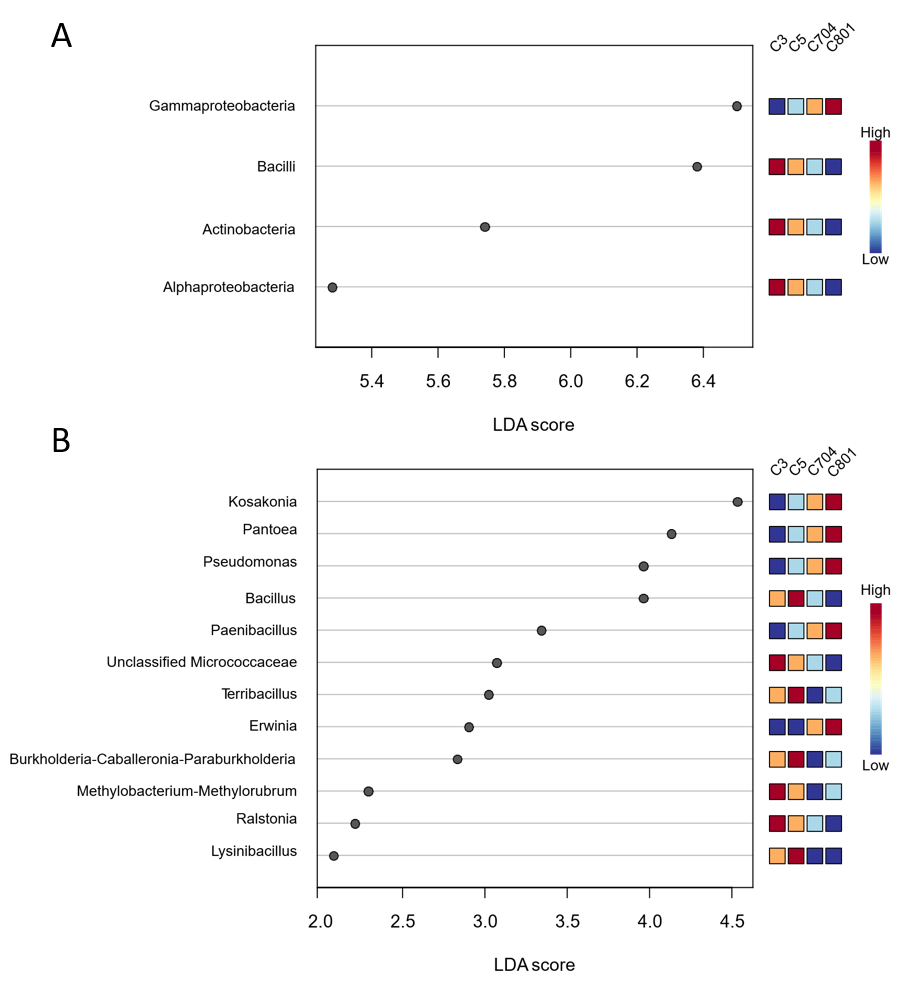


Figure S9: LEfSe analysis indicating differential abundant bacterial taxa between four breeding cycles originating from TLI at (A) class, and (B) genus level . Only bacterial taxa with a LDA score >2 and a P < 0.05 are shown in the figure.


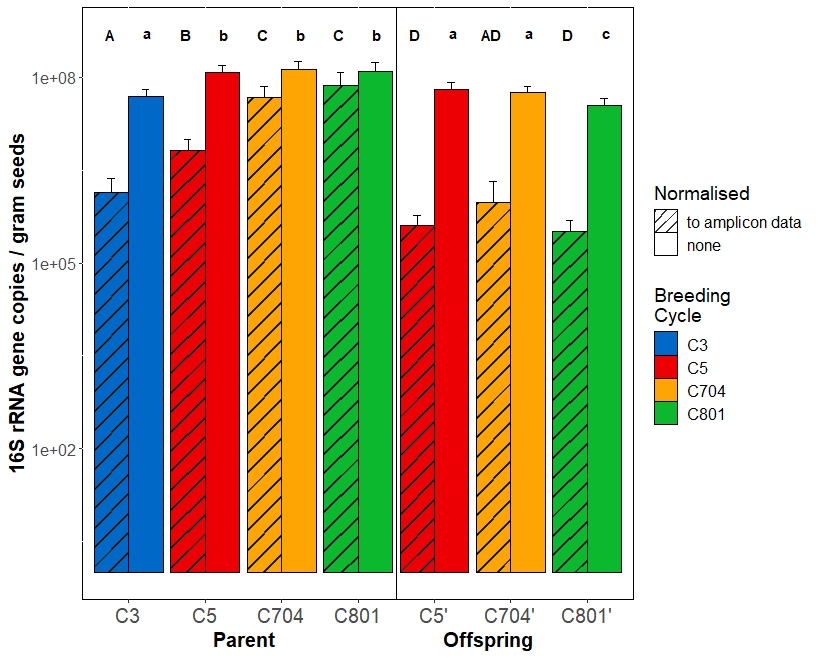


Figure S10: Bacterial abundance per gram seed and in addition normlised to the percentage of host DNA inferred from 16S rRNA gene amplicon sequencing. Significant differences (P< 0.05) were determined by Kruskal-Wallis pairwise test and are indicated by letters.

**Supplementary References**

1. Kolia-Diafouka P, Godreuil S, Bourdin A, Carrère-Kremer S, Kremer L, van de Perre P, Tuaillon E. Optimized lysis-extraction method combined with IS6110-amplification for detection of Mycobacterium tuberculosis in paucibacillary sputum specimens. Frontiers in Microbiology. 2018;9:2224.

2. Ortega RA, Mahnert A, Berg C, Müller H, Berg G. The plant is crucial: specific composition and function of the phyllosphere microbiome of indoor ornamentals. FEMS Microbiology Ecology. 2016;92:fiw173.

3. Hall TA. BioEdit: a user-friendly biological sequence alignment editor and analysis program for Windows 95/98/NT. In: : Oxford. p. 95–98.

4. Camacho C, Coulouris G, Avagyan V, Ma N, Papadopoulos J, Bealer K, Madden TL. BLAST+: architecture and applications. BMC bioinformatics. 2009;10:1–9.

5. Flemer B, Gulati S, Bergna A, Rändler M, Cernava T, Witzel K, et al. Biotic and abiotic stress factors induce microbiome shifts and enrichment of distinct beneficial bacteria in tomato roots. Phytobiomes Journal. 2022;6:276–89.

6. Pinzon NM, Ju L-K. Improved detection of rhamnolipid production using agar plates containing methylene blue and cetyl trimethylammonium bromide. Biotechnology letters. 2009;31:1583–8.

7. Gordon SA, Weber RP. Colorimetric estimation of indoleacetic acid. Plant physiology. 1951;26:192.

8. Weinert N, Meincke R, Gottwald C, Heuer H, Schloter M, Berg G, Smalla K. Bacterial diversity on the surface of potato tubers in soil and the influence of the plant genotype. FEMS Microbiology Ecology. 2010;74:114–23.
